# Potential effects of carbon monoxide donor and its nanoparticles on experimentally induced gastric ulcer in rats

**DOI:** 10.1007/s10787-023-01166-4

**Published:** 2023-03-08

**Authors:** Alaa E. Elsisi, Esraa F. Mekky, Sally E. Abu-Risha

**Affiliations:** Pharmacology and Toxicology Department, Faculty of Pharmacy, Tanta University, Tanta, Egypt

**Keywords:** Carbon monoxide, CORM2, COX, Gastric ulcer, HO-1, NRF2, Oxidative stress

## Abstract

The prevalence of gastric ulcers is increasing worldwide, especially those brought on by non-steroidal anti-inflammatory drugs (NSAIDS), so prevention is extremely crucial. The protective potential of carbon monoxide (CO) in several inflammatory disorders has been clarified. The goal of the current study was to investigate the gastroprotective effect of CO produced by its pharmacological donor (CORM2) and its nanoparticles (NPs) against indomethacin (INDO)-induced ulcers. Investigations on CORM2's dose-dependent effects were also conducted. For induction of gastric ulcer, 100 mg kg^−1^ of INDO was given orally. Before ulcer induction, CORM2 (5, 10, and 15 mg kg^−1^), CORM2 nanoparticles (5 mg kg^−1^), or ranitidine (30 mg kg^−1^) were given intraperitoneally for 7 days. Ulcer score, gastric acidity, gastric contents of malondialdehyde (MDA), nitric oxide (NO), heme oxygenase-1 (HO-1), and carboxyhemoglobin (COHb) blood content were estimated. Additionally, gene expression of nuclear factor erythroid 2-related factor 2 (NRF2) and immunohistochemical staining of cyclooxygenase-1 (COX-1) as well as cyclooxygenase-2 (COX-2) were analyzed. Results demonstrated a substantial dose-dependent decrease in ulcer score, pro-inflammatory indicators, and oxidative stress markers with CORM2 and its NPs. Furthermore, CORM2 and its NPs markedly increased NRF2, COX-1, and HO-1, but CORM2 NPs outperformed CORM2 in this regard. In conclusion, the CO released by CORM2 can protect against INDO-induced gastric ulcers dose dependently, and the highest used dose had no effect on COHb concentration.

## Introduction

One of the most common conditions affecting the upper gastrointestinal system is gastric ulcer, a condition that affects people worldwide and is best defined as a stomach lesion, its prevalence ranges from 2.4 to 6.07% (Bi et al. 2014). Gastric ulcers can occur by varied reasons, including *Helicobacter pylori*, alcohol abuse, pathogens, and NSAIDS. These variables collectively increase the amount of reactive oxygen species (ROS) in the stomach, resulting in oxidative stress (Suzuki et al. 2012).

Although its physiological effects on the human body were first recognized in the early twentieth century, CO has long been regarded as a silent killer (Motterlini and Otterbein 2010). CO is an endogenously produced gaseous intermediate, generated from heme degradation by enzyme heme oxygenase, which also generates free iron and biliverdin (Bannenberg and Vieira 2009). It exhibits anti-inflammatory, antiapoptotic, antihypertensive, vasodilator, and cytoprotective effects at low concentrations (Ling et al. 2018), via activation of p38 mitogen-activated protein kinase (p38MAPK) signaling pathway, interacting with hemoproteins, i.e., COX-1, COX-2, and other cellular targets such as NRF2 and HO-1 (Amersi et al. 2002; Wang et al. 2011; Magierowska et al 2018a).

Transition metal carbonyl complexes known as carbon monoxide releasing molecules (CORMs) release CO into the body in a controlled release manner without increasing the quantity of carboxyhemoglobin generated over the normal level. Additionally, this gives them an advantage over the use of inhaled CO gas in medicine, which lacks the ability to be controlled released or targeted to specific tissues, has a low solubility in water and body fluids, implying that a high gas concentration may need to be inhaled to produce a desired effect (Ismailova et al. 2018). CORMs consist of a transition metal core, i.e., ruthenium (CORM2, CORM3), manganese (CORM1), or iron (CORM-F3) surrounded by carbonyl groups as a coordinated ligand. The release of CO from CORMs is triggered by several mechanisms such as ligand exchange, enzyme release, and photo-induced release (Schatzschneider 2015). Interestingly, studies showed that the pharmacological donor tricarbonyldichlororuthenium (II) dimer (CORM2) can accelerate the healing of gastric ulcers and shield the gastric mucosa from injury brought on by exposure to systemic stress, the use of alcohol, alendronate, or aspirin administration (Magierowska et al. 2015, 2016; Magierowski et al. 2016b, 2017, 2018). CORM2 has a too short half-life and poor water solubility despite the regulated and spontaneous release of CO (Motterlini 2007; Foresti et al. 2008; Kautz et al. 2016). To improve the CORM2 solubility, tissue targeting, controlled release properties, therapeutic performance, and even bioavailability, many approaches have been developed including micelles, copolymers, and nanoparticles (Faizan et al. 2019).

The controlled release properties, drug protection from the environment, increase in bioavailability, and a high therapeutic index of polymeric nanoparticles, which vary in size from 1 to 1000 nm, have newly spotlighted (Zielinska et al. 2020). Enhanced circulation time, decreased aggregation, prolonged drug delivery, decreased binding to non-target serum and tissue proteins, and increased solubility in buffer and serum are all the effects resulting from adding non-toxic polyethylene glycol (PEG) to the nanoparticle surface (Zhang et al. 2014; Suk et al. 2016; Shi et al. 2021). PEG can be utilized as a capping and stabilizing agent in nanotechnology due to its chemical structure, which consists of hydrophilic oxygen and hydrophobic ethylene units (Derkaoui et al. 2007; Javed et al. 2020). Although CORM2 has been shown to be beneficial in the prevention of gastric ulcers in prior studies, its dose-dependent effect and the underlying mechanisms are still being investigated. Its low water solubility and excessively brief half life are further barriers to using it as a therapeutic agent. Herein, pegylated CORM2 nanoparticles were fabricated in an effort to increase CORM2's bioavailability, solubility, controlled release properties, half life, and circulation time. We investigated whether CORM2 nanoparticles could enhance CORM2's effect in prophylaxis against INDO-induced ulcers. We also clarified the CORM2's dose-dependent protective impact. The mechanisms of the potential protective effect of CO released from its pharmacological donor CORM2 against INDO-induced ulcer were also investigated.

## Materials and methods

### Material

CORM2 was bought from Sigma-Aldrich, USA. INDO was got as a gift from Cairo pharmaceutical Ind., (Cairo, Egypt) and ranitidine hydrochloride was used as solution for injection (Rani, 25 mg mL^−1^) from Pharco pharmaceuticals Ind., (Cairo, Egypt). Peg 400 and dimethyl sulfoxide were bought from Sigma-Aldrich, USA. All solutions and nanoparticle suspension were freshly prepared.

### Preparation of CORM2 nanoparticles

CORM2 NPs were synthesized using a method adapted from Abdellah et al. (2018). Synthesis of CORM2 NPs were prepared by dissolving 49.5 mg of CORM2 in 5 mL of acetone, the solution was then added dropwise to 11 mL aqueous solution of PEG. The mixture was stirred under ultrasonication at room temperature for 1 h. The resulting suspension was stirred at 200 rpm at room temperature for 6 h for complete evaporation of the organic solvent.

### Physicochemical characterization of nanoparticles

#### Transmission electronic microscopy (TEM)

Morphology and average particle size of CORM2 PEG NPs were determined by TEM (JEM-ARM200F; JEOL, USA). The sample was dried, and then it was analyzed at an acceleration voltage of 200 kV after being spread onto carbon film coated copper grid (200 meshes).

#### Infrared spectroscopy (IR)

The functional groups of CORM2 NPs were identified by Fourier transform infrared spectroscopy (FT-IR) using ATR measurement system (Shimadzu-IRTracer-100/ 206–30,000-39, JAPAN).

#### Zeta potential

Laser Doppler Micro-electrophoresis technique was used to determine the mean zeta potential using Zetasizer Nano ZS (Malvern, UK).

#### Entrapment efficiency

The entrapment efficiency (EE) of CORM2 PEG NPs was calculated indirectly by measuring ruthenium amount in CORM2 solution using iCAP inductively coupled plasma mass spectrometer (iCAP Q ICP-MS, Thermoscientific, USA) according to the assay adapted from (Joshi et al. 2019; El-Sisi et al. 2020b). The same amount of CORM2 PEG NPs sample was then centrifuged at 18,000 rpm for 1 h to precipitate the particles and quantify the ruthenium in the supernatant using the same instrument. The entrapment efficiency of CORM2 PEG NPs was calculated by the following equation:

$$\% {\text{EE}} = {\text{Total}}\;{\text{drug}}-{\text{free}}\;{\text{drug}}/{\text{Total}}\;{\text{drug}} \times 100.$$

CORM2 NPS were stored in dry and sealed glass tube and evaluated for storage stability at 25 °C after 28 days. Samples were collected for monitoring changes in zeta potential and entrapment efficiency at the day 28.

#### Animals

Male albino rats weighting 150–180 g were obtained from the National Research Center (Giza, Egypt). They were housed in the same conditions in wire cages with a 12-h light:dark cycle, 60–70% relative humidity. They were fed standard pellet chow (EL-Nasr Chemical Company, Cairo, Egypt) and free access to water was allowed. They were housed (Faculty of Pharmacy, Tanta University, Egypt) for one week prior to the experiment for acclimatization. This study was conducted according to the ethical principles for the care and the use of laboratory animals confirmed by the Research Ethics Committee, Faculty of Pharmacy, Tanta University (TP/RE/10/22p-0058).

### Experimental design

Rats were randomly distributed into 8 groups each consisting of 8 animals as follows:

Group **1**: Normal control group, Group **2**: Normal control group received CORM-2 (15 mg Kg^−1^, I.P), Group **3**: Gastric ulcer group, Group **4**: Gastric ulcer + ranitidine (30 mg Kg^−1^, I.P.) (El-Sisi et al. 2020a), Group **5**: Gastric ulcer + CORM-2 (5 mg Kg^−1^, I.P), Group **6**: Gastric ulcer + CORM-2 (10 mg Kg^−1^, I.P.) (Magierowski et al. 2017), Group **7**: Gastric ulcer + CORM-2 (15 mg Kg^−1^, I.P.), Group **8**: Gastric ulcer + CORM-2 nanoparticles (5 mg Kg^−1^, I.P.).

Induction of ulcer was achieved by a single oral dose of INDO (100 mg Kg^−1^) (El-Ashmawy et al. 2016). CORM-2, CORM-2 nanoparticles and ranitidine were given daily for 7 days, and in day 7 INDO was administered 1 h following the last dose of each drug. Rats were fasted overnight and deprived of water 2 h before INDO administration. Rats were then anesthetized by ether and euthanized by cervical dislocation 4 h after INDO administration. After that, whole blood samples were collected in heparinized tubes to determine COHb content. Stomachs were excised and opened from the greater curvature, gastric juice was collected in tubes and stomach tissues were rinsed with normal saline. After that, 2 stomachs from each group were randomly selected for histopathological and immunohistochemical examination. The leftover stomachs were divided into weighted pieces and kept at -80 °C for use in the various assays outlined below.

### Measurement of gastric pH

Stomachs were opened through the greater curvature, gastric juice was collected in centrifuge tubes, and then centrifuged at 3000 rpm for 10 min at 4 °C, the clear supernatant (1 mL) was diluted with 1 mL of distilled water, and pH was then measured using a pH meter (Hanna Instruments, Bucharest, Romania) (Sabiu et al. 2016).

### Determination of ulcer score and ulcer index (UI)

The mean number of ulcers per stomach per rat in all groups was calculated as a representative of ulcer score, it was evaluated by a magnification lens in a blinded manner after removing of gastric contents. The UI was estimated by multiplying the ulcer score of each group × 100 based on ulcer score values. The net preventive index was determined as follows: 100% (UI of ulcer group − UI of drug treated group)/UI of ulcer group (El-Ashmawy et al. 2016; El Mahdy et al. 2020).

### Preparation of gastric tissue for assay

The tissue homogenate was prepared for determination of MDA, NO, and HO-1 as follows: 250 mg of gastric tissue was washed with 0.9% normal saline and homogenized in a ice cold phosphate buffer (2.5 mL, pH 7.5) by a PT 3100 Polytron homogenizer (Kinematica instruments, Lucerne, Switzerland). It was then centrifuged at 3000 rpm for 20 min and the supernatant was then isolated for assay.

The gastric contents of MDA and NO were colorimetrically determined using the biodiagnostic kits (Cairo, Egypt) following the manufacturer’s protocols. The HO-1 was determined using ELISA kit provided by sunRed Biological Technology (China) following the manufacturer’s protocol. Calculations were done using the liner regression equation of HO-1 standard curve.

### Determination of NRF2 expression level by qRT–PCR

The level of NRF2 expression was assayed by a quantitative real-time polymerase chain reaction. First, the extraction of total RNA was done using total RNA purification kit according to the manufacturer’s protocol (Thermo Scientific, USA). The extracted RNA analyzed for quantity and quality (A260/A280) and it was then used for cDNA synthesis using QuantiTect reverse transcription (QIAGEN, Germany). After that, the amplification of synthesized cDNA was performed by Maxima SYBR Green/ROX qPCR Master Mix (2X) following the manufacturer’s protocol (Thermo Scientific, USA). The first denaturation step of the PCR cycle was performed at 95 °C for 10 min. Then, using a PCR equipment (QIAGEN, rotor gene 5 plex), 40 denaturation cycles at 95 °C for 15 s, followed by 30 s of annealing at 60 °C, and 30 s of elongation at 72 °C. The housekeeping gene, glyceraldehyde-3-phosphate dehydrogenase (GAPDH), was used as a normalizer for the target gene expression, which was represented as a fold change in relation to the calibrator, using the 2^−∆∆Ct^ method (Livak and Schmittgen 2001). The used primer sequences were NRF2 5-GAGACGGCCATGACTGAT-3(forward), 5–GTGAGGGGATCGATGAGTAA–3(reverse). GADPH 5–AGGTTGTCTCCTGTGACTTC–3(forward), and 5–CTGTTGCTGTAGCCATATTC–3(reverse) (Ueda et al. 2008).

### Measurement of COHb content

The COHb was determined by a multi-component spectrophotometric method using the whole blood samples according to (Attia et al. 2015, 2019). Briefly, 15 uL of blood samples was added to 5 mL distilled water, mixed vigorously, and centrifuged at 10,000 rpm for 10 min. 4 mL of supernatant was put into water bath for 10 min at 35 °C. Then, the absorbance was measured at 4 wavelengths (*λ* = 500, 568, 576, and 630 nm) using a cary UV/VIS double-beam spectrophotometer.

### Histopathological and immunohistochemical analyses

The stomach samples were fixed in 10% neutral buffered formalin, processed in ascending grades of alcohol, cleared in xylene and finally in paraffin to form wax blocks. Thereafter, they were mounted on glass slides and allowed to be stained with hematoxylin and eosin after randomly sectioned to 5 µm thickness for histopathology (Ateufack et al. 2015). Subsequently, the stained slides were evaluated under a light microscope. For immunohistochemical analysis, immunoperoxidase staining was done. The slides were evaluated by a blinded certified pathologist (Beam et al. 2003). COX-1 and COX-2 staining was determined by a semi-quantitative assessment that depends on the percentage of positive cells and also the intensity of staining. We evaluated five 10X fields from all slides. The percentage was estimated as follows: 0 for negative; 1 for < 10% of positive cells; 2 for 10–30%; 3 for 31–60%; 4 for > 60%. The intensity was estimated as follows: 0 for negative; 1 for weak staining; 2 for moderately intense staining; and 3 for strong intense staining. We estimated the final expression score by the multiplication of the intensity with percentage, and then it was classified as weak for scores 1–2, moderate for scores from 3–5, marked for scores from 6–8, and very marked for scores > 9 (Beam et al. 2003).

### Statistical analysis

Data were statistically analyzed by GraphPad prism 5.0 Demo (GraphPad Software, San Diego, CA). The acceptable level of significance was at *P* < 0.05. One-way analysis of variance (ANOVA) was employed for statistical comparison among groups, followed by Tukey, multiple comparison tests. Values are expressed as mean ± SD.

## Results

### Characterization of nanoparticles

#### Particle size and zeta potential

The CORM2 PEG NPs have a mean particle size of 30.1 ± 9.05 nm, according to the TEM measurements of particle size. The zeta potential of CORM2 PEG NPs was 2.47 ± 3.48 mV. The CORM2 PEG NPs system is stabilized by the steric impact of PEG even if the zeta potential is close to zero (Locatelli and Franchini 2012).

#### Entrapment efficiency

The amount of ruthenium was measured by ICP-MS as mentioned above, and the entrapment efficiency was calculated to be 77.4%. CORM2 NPs were evaluated for storage stability at 25 °C in terms of zeta potential and EE% at day 28. Non-significant change was observed for these parameters (2.2 ± 2.84 and 76.3%, respectively), referring to the CORM2 NPs stability at 25 °C for 28 days. Furthermore, no change in appearance was observed after this time.

#### TEM and FT-IR

**Fig. 1:**
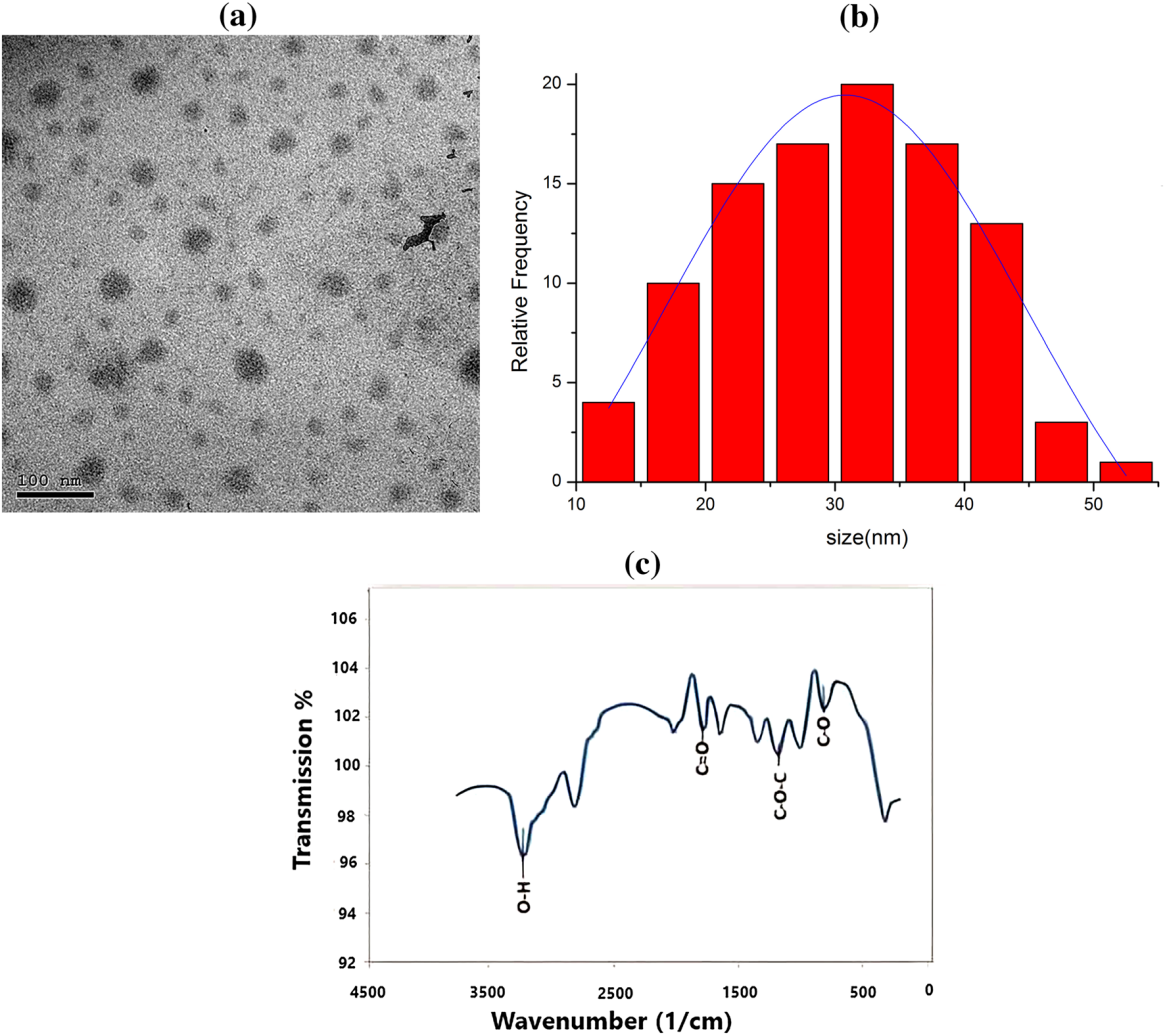
**a** TEM image, **b** histogram of CORM2 nanoparticles and **c** FTIR of CORM2 NPs

The morphology of CORM2 PEG NPs was furtherly confirmed by TEM, which revealed uniform, spherical particles with a gray shadow (Fig. 1a,b). FT-IR revealed CO stretching peaks between around 1800 and 2100 cm^−1^ (Fig. 1c), noting that CORM2 had no break down throughout the preparation (Ramanathan et al. 2021).

#### Effect of different drugs on gastric pH

**Fig. 2:**
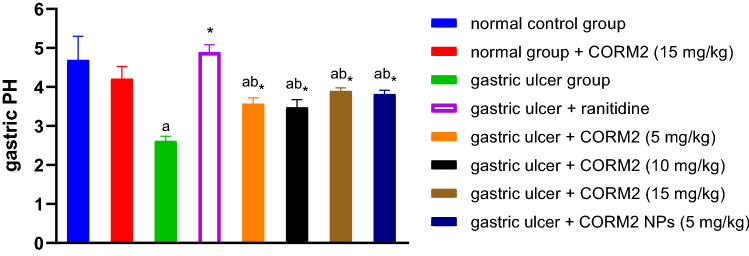
Effect of different drugs on gastric pH. The results are presented as mean ± SD, *n* = 6/group, *p* < 0.05. a A significant difference compared to the normal control group. *Indicates a significant difference compared to the ulcer group. b A significant difference compared to the ranitidine group

INDO administration significantly decreased the gastric pH by about 44.13% relative to the normal control group. Administration of CORM2 in doses of 5, 10, and 15 mg kg^−1^ significantly elevated the pH by about 36.14, 32.74, and 48.66%, respectively relative to the INDO group. Administration of CORM2 NPs significantly elevated pH by 45.53% as compared to the INDO group. In addition, ranitidine significantly elevated the pH by 86.75% relative to the INDO group, and CORM2 15 mg kg^−1^ given to the normal rats did not affect the gastric pH compared to the normal control group (Fig. 2).

#### Effect of different drugs on ulcer index

A high ulcer score of 27.17 ± 1.7 and an ulcer index of 2717 were revealed by INDO administration. In comparison to the INDO group, the ranitidine group had an extremely high preventative value of 95.2% and a low ulcer index of 130. In contrast to the INDO group, pretreatment with CORM2 5, 10 mg kg^−1^ clearly resulted in a significant drop in ulcer score and an increase in preventative index (72.4 and 76.1%, respectively). Compared to the INDO group, CORM2 at 15 mg kg^−1^ and NPs at 5 mg kg^−1^ showed a significantly greater elevation of the net preventative index (90.2 and 95.2%, respectively) and a markedly significant decrease in the ulcer index (266 and 130, respectively) (Table 1).

**Table1:** Effect of different drugs on ulcer score, index and preventive index

Groups	Ulcer sore	Ulcer index	Net preventive index
Normal control group	–	–	–
Normal group + CORM-2 (15 mg per Kg)	–	–	–
Gastric ulcer group	27.17 ± 1.7	2717	0
Gastric ulcer + ranitidine	1.3 ± 0.514*	130	95.2%
Gastric ulcer + CORM-2 (5 mg per kg)	7.5 ± 3.2^b^*	750	72.4%
Gastric ulcer + CORM-2 (10 mg per kg)	6.5 ± 2.4^b^*	650	76.1%
Gastric ulcer + CORM-2 (15 mg per kg)	2.66 ± 1.2^cd^*	266	90.2%
Gastric ulcer + CORM-2 NPs (5 mg per kg)	1.3 ± .5^c^*	130	95.2%

The results are presented as mean ± SD, *n* = 6 /group, *p* < 0.05. *Indicates a significant difference compared to the ulcer group. b A significant difference in comparison to the ranitidine group. c A significant difference in comparison to the gastric ulcer + CORM2 (5 mg kg^−1^) group. d A significant difference relative to gastric ulcer + CORM2 (10 mg kg^−1^).

#### Effect of different drugs on MDA content

**Fig. 3:**
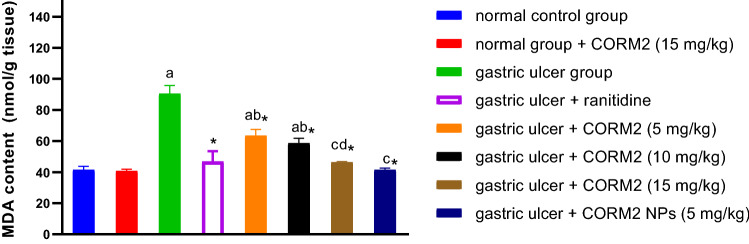
Effect of different drugs on MDA content: The results are presented as mean ± SD, *n* = 6/group, *p* < 0.05. **a** A significant difference in comparison to the normal control group. *Indicates a significant difference in comparison to the gastric ulcer group. **b** A significant difference in comparison to gastric ulcer + ranitidine group. **c** A significant difference compared with gastric ulcer + CORM2 (5 mg kg^−1^) group. **d** A significant difference compared with gastric ulcer + CORM2 (10 mg kg^−1^) group

INDO administration significantly elevated MDA content by 118.02% as compared to the normal control group. Ranitidine administration significantly decreased MDA content by 48.23% relative to the INDO group. We also noted that pretreatment with CORM2 5, 10, and 15 mg kg^−1^ significantly decreased MDA content by 29.83, 35.22, and 48.7%, respectively relative to the INDO group. In addition, pretreatment with CORM2 NPs 5 mg kg^−1^ significantly decreased MDA content as compared to the INDO group by 54.17%. It is shown that CORM2 15 mg kg^−1^ administration to normal rats had no impact on the MDA content as compared to the normal control group (Fig. 3).

#### Effect of different drugs on NO content

**Fig. 4:**
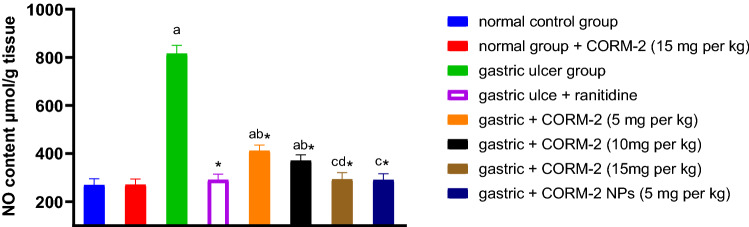
Effect of different drugs on the NO level: The results are presented as mean ± SD, *n* = 6/group, *p* < 0.05. **a** A significant difference in comparison to the normal control group. *Indicates a significant difference in comparison to the gastric ulcer group. **b** A significant difference in comparison to the gastric ulcer + ranitidine group. **c** A significant difference compared with the gastric ulcer + CORM2 (5 mg kg^−1^) group. **d** A significant difference compared with the gastric ulcer + CORM2 (10 mg kg^−1^) group

It is obvious that INDO administration significantly elevated NO content by 203.57% as compared to the normal control group. Ranitidine administration significantly decreased NO content by 64.3% relative to the INDO group. We also found that pretreatment with CORM2 5, 10, 15 mg kg^−1^ significantly decreased NO content by 49.49, 54.59, and 64.08%, respectively compared to the INDO group. Pretreatment with CORM2 NPs 5 mg kg^−1^ significantly decreased NO content as compared to INDO group by 64.37%, but the dose of 15 mg kg^−1^ to the normal rats had no effect on the NO content as compared to the normal control group (Fig. 4).

#### Effect of different drugs on HO-1 concentration

**Fig. 5:**
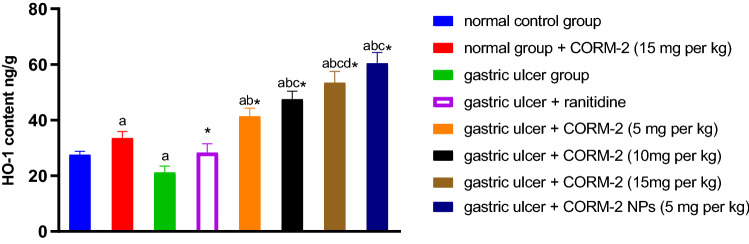
Effect of different drugs on the HO-1 concentration: The results are presented as mean ± SD, *n* = 6/group, *p* < 0.05. **a** A significant difference in comparison to normal control group. * Indicates a significant difference in comparison to the ulcer group. **b** A significant difference in comparison to the ranitidine group. **c** A significant difference in comparison to the gastric ulcer + CORM2 (5 mg kg^−1^) group. **d** A significant difference in comparison to the gastric ulcer + CORM2 (10 mg kg^−1^) group

HO-1 content was significantly lower in the INDO group compared to the normal control group by 23.04%. When compared to the INDO group, ranitidine treatment significantly increased HO-1 levels by 33.44%. 15 mg kg^−1^ of CORM2 administered to normal rats significantly increased the HO-1 concentration by 21.6% as compared to the normal control group. It was shown that administering CORM2 at various doses of 5, 10, and 15 mg kg^−1^ and NPs at a dose of 5 mg kg^−1^ significantly increased HO-1 concentration by 95.05, 123,9, 152.03, and 184.9%, respectively, compared to the INDO group (Fig. 5).

#### Effect of different drugs on NRF2 content

**Fig. 6:**
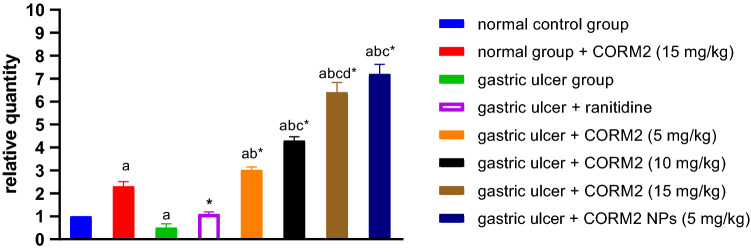
Effect of different drugs on NRF2 expression: The results are presented as mean ± SD, *n* = 6/group, *p* < 0.05. **a** indicates a significant difference in comparison to the normal control group. *Indicates a significant difference in comparison to the ulcer group. **b** A significant difference in comparison to the ranitidine group. **c** A significant change in comparison to the gastric ulcer + CORM2 (5 mg kg^−1^) group. **d** A significant difference in comparison to the gastric ulcer + CORM2 (10 mg kg^−1^) group

In comparison to the normal control group, the INDO group had a significant 50% drop in NRF2 concentration. Ranitidine administration caused 120.4% significant increase in NRF2 content as compared to the INDO group. 15 mg kg^−1^ of CORM2 administered to normal rats significantly increased the NRF2 concentration by 130% compared to the control group. In comparison to the INDO group, CORM2 treatment at doses of 5, 10, and 15 mg kg^−1^ and NPs at 5 mg kg^−1^ significantly increased NRF2 content by 503.6%, 760%, 1180%, 1340%, respectively (Fig. 6).

#### Effect of different drugs on COHb level

**Fig. 7:**
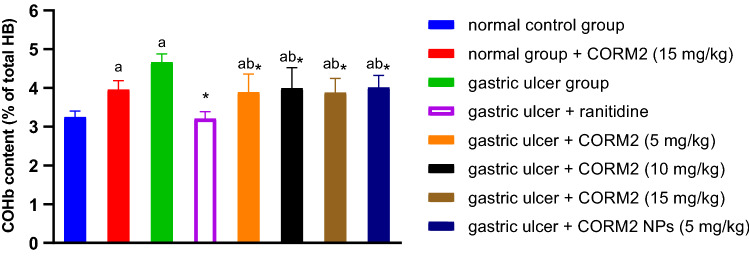
Effect of different drugs on the COHb level: The results are presented as mean ± SD, *n* = 6/group, *p* < 0.05. **a** A significant difference in comparison to the normal control group. *Indicates a significant difference in comparison to the ulcer group. **b** A significant difference in comparison to the ranitidine group

It is interesting to note that INDO group significantly elevated the COHb level by about 43.57% compared to the normal control group. Ranitidine administration significantly decreased COHb level by 31.23% relative to the INDO group, and CORM2 in doses of 5, 10, and 15 mg and CORM2 NPs significantly decreased the COHb level by 16.55, 14.38, 16.78, and 13.95%, respectively, compared to the INDO group. CORM2 15 mg kg^−1^ in normal rats significantly elevated the COHb level by 21.84% relative to the normal control group (Fig. 7).

#### Effect of different drugs on histopathological examination

**Fig. 8:**
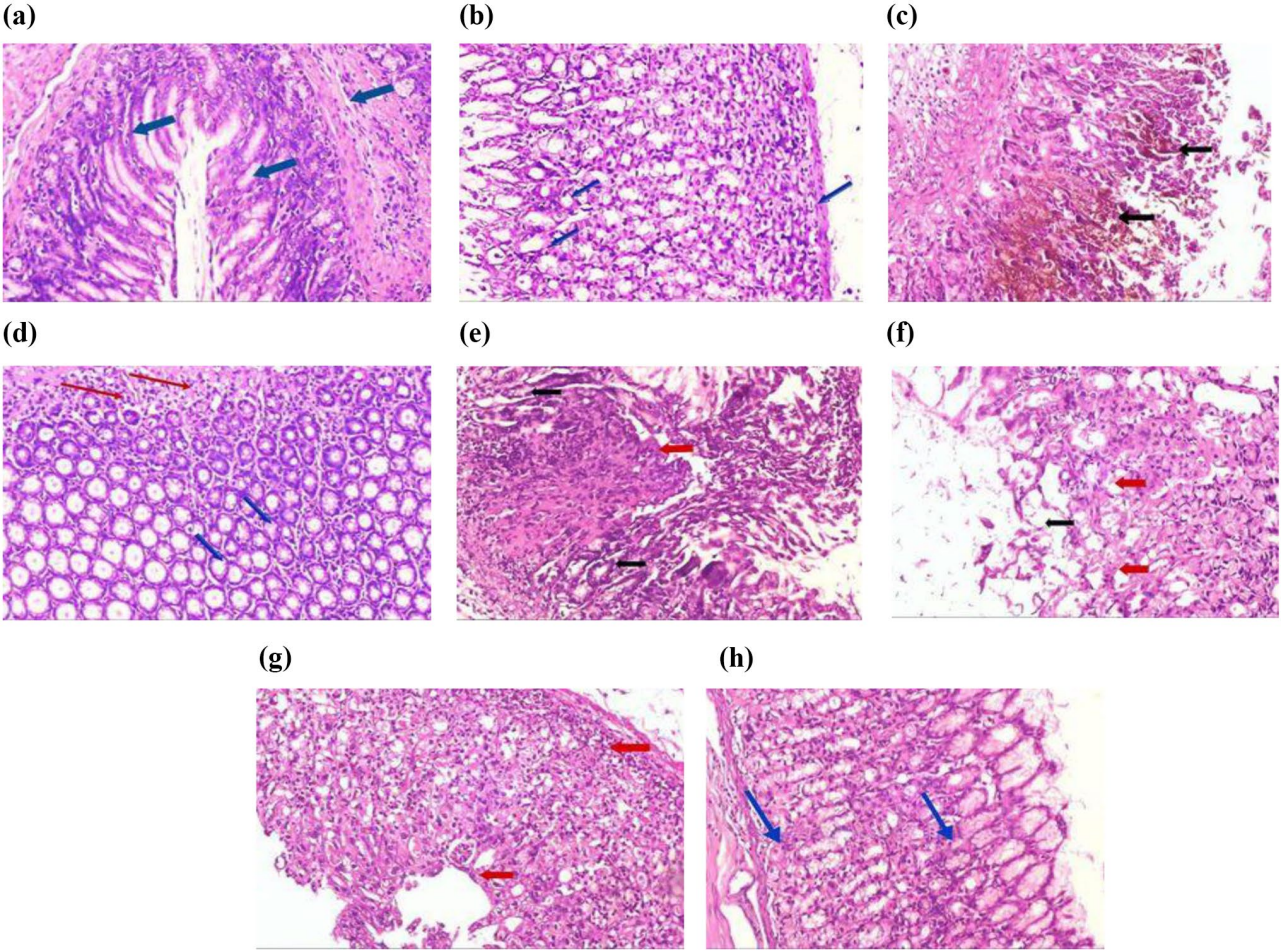
**a** Normal control group showing normal gastric mucosal glands consisting of glands lined with columnar mucin secreting cells surrounded by normal musculosa (*blue arrows*) (H&E ×100). **b** Normal control group + CORM-2 (15 mg kg^−1^) showing: Normal gastric mucosal glands consisting of glands lined with columnar mucin secreting cells surrounded by normal musculosa (*blue arrows*) (H&E ×100). **c** Gastric ulcer group sections showing: Section of stomach showing massive mucosal ulceration with congestion (*black arrows*) (H&E ×100). **d** Gastric ulcer + ranitidine group showing: Section of stomach of rats showing hyperplastic gastric glands with no ulceration (*blue arrow*) surrounded by minimal chronic inflammatory cells (red arrows) (H&E ×100). **e** Gastric ulcer + CORM-2 (5 mg kg^−1^) group showing ulcerated gastric mucosa lined by granulation tissue (*black arrow*) surrounded by few proliferated gastric glands (red arrow) (H&E ×100). **f** Gastric ulcer + CORM-2 (10 mg kg^−1^) group showing: ulcerated gastric mucosa (*black arrow*) surrounded by proliferated gastric glands (*red arrow*) (H&E ×100). **g** Gastric + CORM-2 (15 mg kg^−1^) group showing: A partial healing of the ulcers surrounded by marked proliferated gastric glands with minimal submucosal inflammation (*red arrows*) (H&E ×100). **h** Gastric ulcer + CORM-2 nanoparticles (5 mg kg^−1^) group showing normal thickness of gastric mucosa, no ulceration or inflammation (*blue arrows*) (H&E ×100) (Color figure online)

Gastric sections from INDO group showed ulceration, loss of gastric mucosa, and congestion as compared to the normal control group (Fig. 8c). Sections for CORM2 15 mg kg^−1^ in normal rats showed a normal gastric tissue (Fig. 8b). Ranitidine administration caused hyperplastic gastric glands with no ulceration surrounded by the minimal chronic inflammatory cells (Fig. 8d). Pretreatment of CORM2, 5, and 10 mg kg^−1^ doses showed a lower grade of ulceration and inflammation as compared to INDO group (Fig. 8e, f). We found that the dose of 15 mg kg^−1^ relieved ulcer and inflammation at a higher rate than the lower doses (Fig. 8g). On the other hand, pretreatment with CORM2 NPs 5 mg kg^−1^ showed a normal thickness of gastric mucosa with no ulceration or inflammation as compared to the INDO group (Fig. 8h).

#### Effect on COX-1 immunostaining

**Fig. 9:**
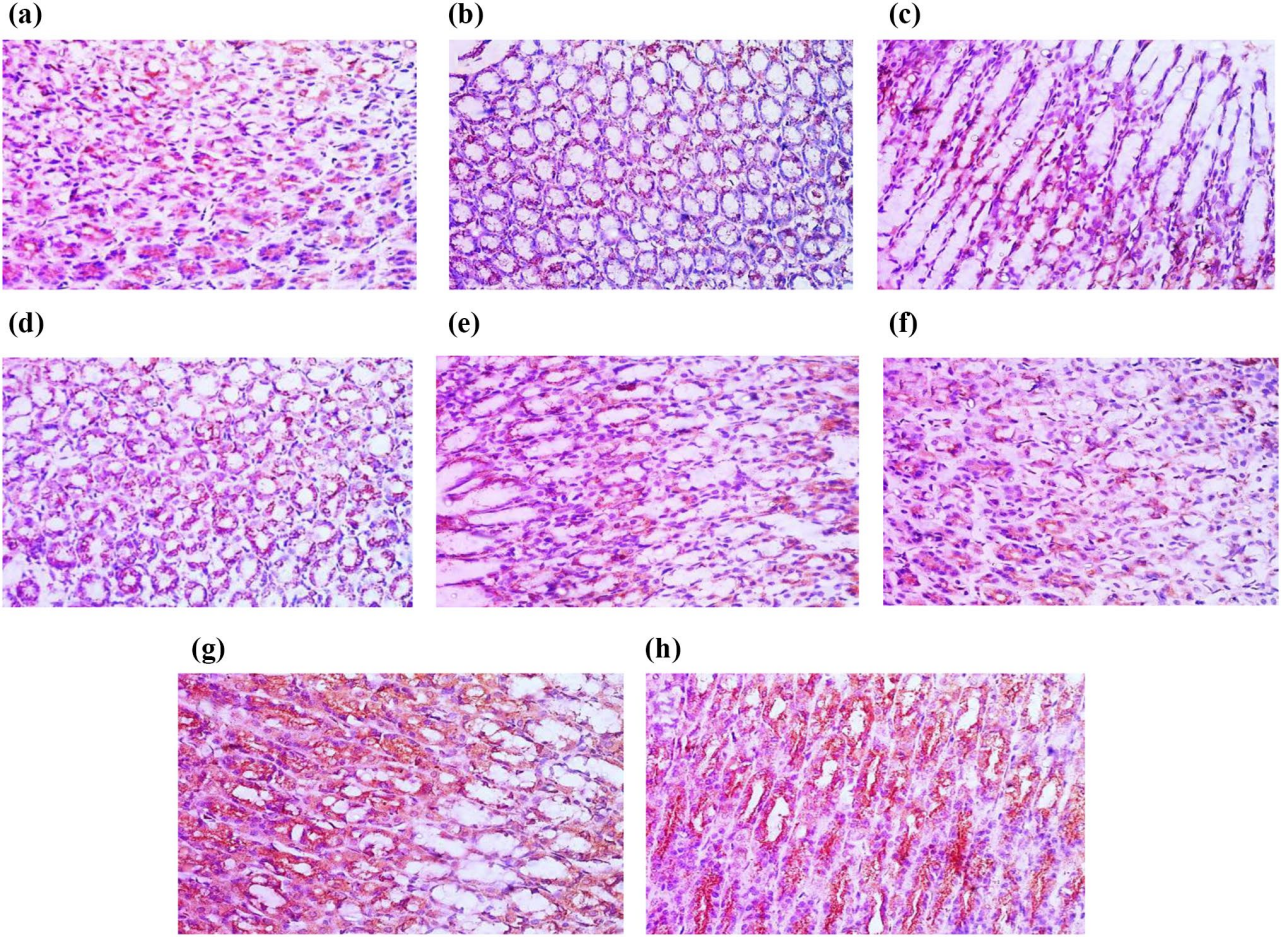
**a** The normal control group showing marked COX-1 expression in about 60% of gastric mucosal glands with a moderate intensity [score 6] (×200), **b** The normal control group received CORM-2 (15 mg kg^−1^) showing marked COX-1 expression in about 50% of gastric mucosal glands with a moderate intensity [score 6] (×200), **c** the gastric ulcer group showing weak COX-1 expression in less than 10% of gastric mucosal glands with a moderate intensity [score 2] (×200), **d** the gastric ulcer + ranitidine group showing a moderate COX-1 expression in about 20% of gastric mucosal glands with a moderate intensity [score 4] (×200), **e** the gastric ulcer + CORM-2 (5 mg kg^−1^) group showing marked COX-1 expression in about 50% of gastric mucosal glands with a moderate intensity [score 6] (×200), **f** the gastric ulcer + CORM-2 (10 mg kg^−1^) group showing marked COX-1 expression in about 40% of gastric mucosal glands with a moderate intensity [score 6] (×200), **g** the gastric ulcer + CORM-2 (15 mg kg^−1^) group showing very marked COX-1 expression in about 60% of gastric mucosal glands with a strong intensity [score 9] (×200), **h** the gastric ulcer + CORM-2 nanoparticles (5 mg kg^−1^) group showing very marked COX-1 expression in about 60% of gastric mucosal glands with a strong intensity [score 9] (×200)

Normal control group with or without administration of CORM2 15 mg kg^−1^ gastric section showed high immunostaining of COX-1 (score 6, Fig. 9a, b). The INDO administration decreased COX-1 immunostaining (score 2) compared to the normal control group (Fig. 9c). Pretreatment with ranitidine and CORM2 in different doses and NPs increased COX-1 immunostaining compared to the INDO group (Fig. 9d–h).

#### Effect on COX-2 immunostaining

**Fig. 10:**
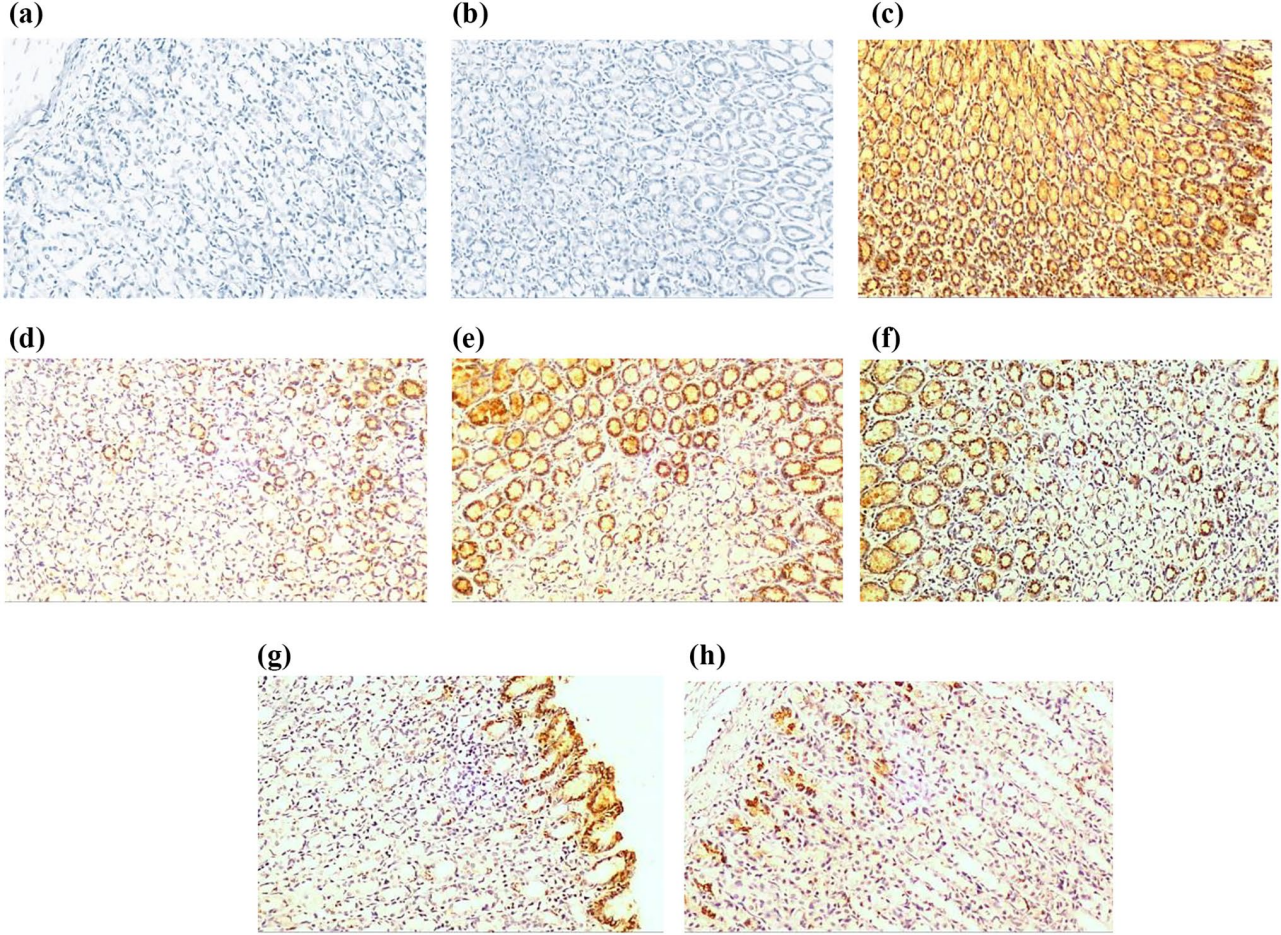
**a** The normal control group showed negative COX-2 expression in gastric mucosal glands [score 0] (×100), **b** the normal control group received CORM-2 (15 mg kg^−1^) showing: negative COX-2 expression in gastric mucosal glands [score 0] (×100), **c** the gastric ulcer group showing very marked COX-2 expression in more than 60% of gastric mucosal glands with a strong intensity [score 12] (×100), **d** the gastric ulcer + ranitidine group showing weak COX-2 expression in less than 10% of gastric mucosal glands with a moderate intensity [score 2] (×100), **e** the gastric ulcer + CORM-2 (5 mg kg^−1^) group showing marked COX-2 expression in about 60% of gastric mucosal glands with a moderate intensity [score 6] (×100), **f** the gastric ulcer + CORM-2 (10 mg kg^−1^) group showing marked COX-2 expression in about 40% of gastric mucosal glands with a moderate intensity [score 6] (×100). **g** the gastric ulcer + CORM-2 (15 mg kg^−1^) group showing moderate COX-2 expression in less than 10% of gastric mucosal glands [only apical glands] with a strong intensity [score 3] (×100), **h** the gastric ulcer + CORM-2 nanoparticles (5 mg kg^−1^) group showing weak COX-2 expression in less than 10% of gastric mucosal glands with a moderate intensity [score 2] (×100)

The normal group and treatment with CORM2 15 mg kg^−1^ in normal rats showed negative immunostaining of COX-2 (Fig. 10a, b). INDO administration increased COX-2 immunostaining and showed a high score (12) compared to control group (Fig. 10c). Ranitidine group showed a weak immunostaining (score 2) (Fig. 10d). Pretreatment of CORM2 5, and 10 mg kg^−1^ showed a marked COX-2 immunostaining of gastric mucosal glands with a moderate intensity (score 6) (Fig. 10e, f). Pretreatment of CORM2 dose of 15 mg kg^−1^ showed a moderate immunostaining (score 3) (Fig. 10g). CORM2 NPs in dose of 5 mg kg^−1^ showed a weak COX-2 immunostaining (Fig. 10h).

## Discussion

The development of gastric ulcers, an inflammatory illness that affects people all over the world, occurred when aggressive and inflammatory mediators overcame the protective factors (Fu et al. 2018). Use of NSAIDS, such as indomethacin, is linked to damage to the gastric mucosa (Athaydes et al. 2019). Based on previous studies, CO is well-known to have a gastroprotective action through its anti-inflammatory, anti-apoptotic, and vasodilator effects (Magierowska et al. 2018a).

It was important to not only study the effect of CORMs, but we also realized the importance of preparing a new form of CORM2 NPs with ECO friendly method to overcome its hydrophobicity and short half life which hinder its clinical utility. Ultrasonication is a novel technique in preparation of nanosuspension (Duan et al. 2015; Jacob et al. 2020). CORM2 NPs were prepared via sonoproduction process in the presence of PEG as a stabilizer (Abdellah et al. 2018; Low et al. 2022). Our results showed that a high proportion of CORM2 was incorporated in the PEG which is characterized by increasing circulation time, decreasing aggregation, thereby improving the bioavailability as mentioned above. This can explains the superiority of CORM2 NPs in reducing the proinflammatory markers and increasing the protective markers as compared to CORM2 solutions.

After INDO administration, stomach sections revealed the development of ulcers, inflammation, and loss of gastric mucosa. Pretreatment with CORM2 at various doses demonstrated a dose-dependent reduction in ulcer and inflammation as indicative of CO gastroprotective efficiency. Furthermore, treatment with CORM2 at the higher dose (15 mg kg^−1^) in normal control rats failed to cause any congestion or inflammation. Additionally, CORM2 NPs had the most protective effects on ulcerated rats, which strongly suggests that NPs are more bioavailable than drug solutions. This finding is well agreed with the ulcer index results which were reduced by different doses of CORM2, whereas the higher dose of CORM2 15 mg kg^−1^ had a better preventive index as compared to lower doses 5 and 10 mg kg^−1^, and the NPs had the lowest ulcer score and highest preventive index. Ranitidine showed high preventive index, due to its H2 blocking effect on parietal cells (MacFarlane 2018), its effect was comparable to CORM2 nanoformulation. This supports the facts that indomethacin damages mucous membranes and forms ulcers through a variety of pathways and that CO produced from its pharmacological donor has a well-known gastroprotective effect (Athaydes et al. 2019; Buttgereit et al. 2001; Magierowska et al. 2018a).

Based on the data, the gastric pH of INDO group was significantly decreased compared with the normal control group. CORM2 solutions and NPs significantly elevated the gastric pH compared to the INDO group. This result can be explained as INDO caused elevation of gastric acidity by free radicals formation or inhibition of prostaglandins (PG) synthesis (Sabiu et al. 2016). The CO which was released from CORM2 reduced the gastric acidity via increasing gastric blood flow, hence promotes ulcer healing and increase prostaglandin E2 synthesis as well (Magierowska et al. 2019), which stimulates bicarbonate and mucus secretion (Takeuchi and Amagase 2018). Ranitidine was superior in reducing gastric acidity which may be due to its direct action on parietal cells, it blocks histamine receptors and thus suppress both stimulated and basal gastric acid secretion produced by histamine (MacFarlane 2018).

Here, we evaluated the MDA concentration as a measure of lipid peroxidation. INDO significantly increased the MDA concentration, and this is in accordance with earlier studies (Harakeh et al. 2022), CORM2 solutions and NPs significantly decreased its content. NPs and higher doses were preferred over the lower doses and their effect on MDA reduction was similar to ranitidine, which previously inhibited lipid peroxidation in indomethacin-induced gastric ulcer model (Sokar, et al. 2016; El-Sisi et al. 2020a). The reduction in MDA content is one of the protective mechanisms for CO, released by CORM2, and is consistent with earlier studies (Magierowski et al. 2018).

NO as well as CO is an endogenous gaseous mediator which has a physiological role in maintaining the gastric mucosal integrity (Wallace et al. 2017). Interestingly, it can also be cytotoxic because of overproduction which result in oxidative stress during the inflammatory processes (Valko et al. 2007). Kim et al. documented that ROS, such as NO, has a role in the pathogenesis of GIT damage and it is released in large amounts in the inflammatory areas (Kim et al. 2012). Indomethacin administration showed to increase NO level in damaged gastric tissue compared to the healthy tissue (Morsy and Fouad 2008). Joshi et al. showed that CORM2 reduced the level of both inducible and endothelial nitric oxide synthase which alleviated mechanical allodynia and mechanical hyperalgesia (Joshi et al. 2019). In our study, NO was significantly elevated by INDO administration and CO released from CORM2 solutions and NPs significantly reduced NO, higher doses and NPs were superior to the lower doses and their effect was comparable to ranitidine, that previously decreased inducible nitric oxide synthase (iNOS) activity which increased by indomethacin administration (Bayir et al. 2006). This result may be confirmed by the observation of Wang et al., where CO exerted a protection against intestinal inflammatory response by inhibition of iNOS and NO production (Wang et al. 2012).

As an attempt to discover the exact mechanism of CO released from CORM2 and its NPs, we focused on NRF2 signaling pathway. El Badawy et al. supposed that NRF2 and its downstream enzymes, such as HO-1, has a beneficial role in the gastroprotection against indomethacin-induced gastric ulcer (El Badawy et al. 2021), also other studies showed that HO-1 induction has a beneficial role in NSAIDS-induced gastric damage (Lee et al. 2012), well agreed with our study that showed a significant reduction in NRF2 and HO-1 levels after INDO administration compared to the control group. Pretreatment with CORM2 and its NPs caused a significant elevation in NRF2 as well as HO-1 contents compared to the INDO group, CORM2 NPs was superior in enhancing the expression of NRF2 and HO-1 content compared to the CORM2 solution. Ranitidine significantly elevated NRF2 and HO-1 compared to INDO group, this agrees with previous studies (Keshk et al. 2017), but its effect was inferior to CORM2. Additionally, in agreement with Magierowski et al., we may draw the conclusion that the NRF2/HO-1 pathway plays a crucial role in gastroprotection against INDO injury and represents a potential key mechanism of the protective and healing impact of CO produced by CORM2 against the gastric damage (Magierowski et al. 2017).

COX inhibition is a key mechanism of NSAIDS-induced gastric ulcer and side effects. It is thought that inhibition of gastroprotective prostaglandins, which are generated mainly by COX-1, is primarily responsible for NSAIDs gastrointestinal side effects (Buttgereit et al. 2001; Al-Saeed 2011). It is important to know that there are other mechanisms for GIT injury which had no relation to prostaglandins such as mitochondrial and cellular injury (Colucci et al. 2009). Inhibition of COX-2, an inducible enzyme that is predominantly expressed during inflammation and is elevated after gastric injury brought on by NSAIDS or other stimuli, is believed to lower the chance of developing ulcers (Jackson et al. 2000; Buttgereit et al. 2001; Fornai et al. 2011; Magierowska et al 2018b; Magierowski et al. 2018). Herein, indomethacin administration elevated COX-2 expression and caused gastric damage, agreed with Shu et al., that demonstrated elevated COX-2 expression in indomethacin-induced rat intestinal damage (Shu et al. 2019). Shaik and Eid mentioned that elevated COX-2 expression may represent a compensatory response to inhibition of prostaglandin biosynthesis after COX-1 inhibition (Shaik and Eid 2022). The elevated COX-2 expression in gastric tissue was significantly decreased by ranitidine, and this agrees with other studies in which ranitidine decreased COX-2 level in ethanol-induced ulcer model (Salman et al. 2019). CORM2 in its various doses significantly decreased COX-2 expression. Therefore, consistent with other studies (Magierowska et al. 2016), we assume that suppressing the proinflammatory COX-2 is a possible mechanism for gastroprotective effect of CO released by CORM2. Contrarily, COX-1 is the constitutive enzyme isoform, and it normally expresses itself in elevated levels in gastric tissue without being influenced by inflammation (Guo et al. 2003). Studies showed that COX-1 expression was downregulated by indomethacin (Sokar et al. 2016; Shu et al. 2019). In this study, pretreatment with CO donor elevated COX-1 expression which was downregulated by indomethacin. As shown in previous studies (Fang et al. 2019), ranitidine elevated COX-1 expression, but at a lower extent than CORM2. This result is in correlation with previous studies which suppose that CO may activate PG/COX system and maintain the production of protective prostaglandins (Magierowska et al. 2015).

When COHb levels in the blood surpass a certain threshold, it results in CO poisoning, thus we assessed COHb levels as a safety marker for CORM2 to ensure that COHb did not exceed the baseline value (≤ 5% COHb) (Ling et al. 2018). In our investigation, indomethacin induced a minor but significant increase in COHb levels compared to the control group, and treatment with CORM2 at various doses considerably lowered those levels. According to Magierowski et al., NSAIDS damaged the gastric capillaries resulting in a loss of endogenous CO, which has been immediately taken up by hemoglobin and led to elevated COHb, and that CORM2 could normalize its level because of maintaining of the vascular bed (Magierowski et al. 2016a). It is important to observe that CORM2 did not elevate the COHb level above the baseline level.

## Conclusion

We draw the conclusion that CO released from its pharmacological donor, CORM2, protects against indomethacin-induced gastric ulcer through a variety of mechanisms, including activation of the NRF2/HO-1 pathway, a decrease in oxidative stress, lipid peroxidation through a reduction of NO and MDA, an increased COX-1, and a decrease in proinflammatory COX-2. Furthermore, it is significant to highlight that CORM2 delivered as nanoparticles outperformed other dosages, demonstrating the increased bioavailability of nanoparticles. The higher dose of CORM2 used maintained COHb at baseline level. Further studies are demanded to examine CORM2 use and efficacy in clinical trials.

## Data Availability

The datasets generated during and/or analysed during the current study are available from the corresponding author on reasonable request.
